# The role of microRNA in the pathogenesis of glial brain tumors

**DOI:** 10.1016/j.ncrna.2022.02.005

**Published:** 2022-02-25

**Authors:** Ozal Beylerli, Ilgiz Gareev, Albert Sufianov, Tatiana Ilyasova, Fan Zhang

**Affiliations:** a Federal Center of Neurosurgery, Tyumen, Russia; b Department of Neurosurgery, Sechenov First Moscow State Medical University (Sechenov University), Moscow, Russia; c Department of Anesthesiology, The First Affiliated Hospital of Harbin Medical University, Harbin, China; d Bashkir State Medical University, Ufa, Republic of Bashkortostan, Russia

**Keywords:** Glioma, Glioblastoma, miRNA, Expression, Pathogenesis, Therapy

## Abstract

Gliomas are the most common and fatal primary brain tumor in adults. Gliomas are highly invasive tumors with the highest mortality among all primary malignant brain tumors. Until now, the molecular mechanism that is responsible for glioma tumorigenesis and progression remains unclear. MicroRNAs (miRNAs) are short non-coding RNAs with 18–22 nucleotides in length which function as key regulators of various biological processes through negative control over gene expression at the post-transcriptional level. MiRNA dysregulation plays a key role in cancer oncogenesis, including the development and progression of gliomas.

MiRNAs regulate a wide range of tumor processes including cell proliferation, differentiation, angiogenesis, invasion and apoptosis. In addition, microRNAs can be selectively packaged, secreted, and transported between cells in exosomes that are able to cross the blood-brain barrier (BBB) and are readily available in almost all types of human body fluids, making them promising biomarkers for gliomas.

Increasing evidence has shown that miRNAs play an important role in glioma. For example, a large number studies indicated that this miRNA-21could affect on a variety of cellular and molecular pathways such as insulin-like growth factor (IGF)-binding protein-3 (IGFBP3), RECK, and TIMP3. Exosomal miR-21 may has key roles in gliomas pathogenesis. These findings indicated that miR-21 has critical roles in gliomas pathogenesis and could be used as diagnostic and therapeutic biomarkers for glioma patients.

Profiling miRNAs expression in various human pathological conditions is a rapidly growing field, and it is likely that the knowledge gained from these studies regarding the genesis of gliomas will have the potential in the field of minimally invasive therapy with miRNA to improve the prognosis of patients with this pathology.

## 1 Introduction

Gliomas are the most common (∼80%) primary tumors of the human central nervous system. According to the World Health Organization (WHO) classification of tumors of the central nervous system (CNS), gliomas have been classified into four main histological groups (classes 1–4) according to their microscopic characteristics (such as cytological atypia, anaplasia, mitotic activity, microvascular proliferation, and necrosis) and clinical manifestations. These tumors were also subdivided into astrocytomas (WHO Grade 1–4 grades), oligodendrogliomas (WHO Grade 2–3) and mixed oligoastrocytomas (WHO Grade 2–3) depending on their putative cell origin [1]. Grade 4 glioma or glioblastoma is the most common and fatal primary brain tumor that varies with age. The highest incidence of anaplastic astrocytomas (WHO Grade 3) and glioblastoma are common among patients over 75–84 years old, but oligodendrogliomas are more common in those aged 35–44 years. Glioblastoma has a very aggressive clinical course with an average survival time of 12.2–18.2 months and less than 5% of patients living after 5 years of the initial diagnosis [[1], [2], [3]]. In contrast, low-grade (WHO Grade II-III) gliomas, which account for about one third of all gliomas, are usually less aggressive tumors with a very variable clinical course, but which are not adequately predicted based on their histological class (see Table 1).

**Table 1:** Phases of biomarker research.

Phases	Research	Description
Phase 1	Preclinical studies	The search includes preliminary research to identify potentially useful biomarkers
Phase 2	Process for developing a clinical analysis for a disease	Clinical analysis validation occurs when the biomarker under study can be used to determine their ability to distinguish between people with a tumor and those without a tumor - a case-control study
Phase 3	Longitudinal retrospective studies	Determines the ability of a biomarker to detect preclinical disease by testing the biomarker on tissues collected in a longitudinal retrospective study from study groups. The level of expression of the biomarker is measured in samples taken from individuals before the diagnosis of the tumor, and compared with the level of expression of the biomarker in age-matched control groups.
Phase 4	Prospective screening studies	Tests should determine whether the biomarker can detect a tumor at an early stage of development. People who are asymptomatic are tested, and those who test positive are monitored to determine if they have a tumor.
Phase 5	Tumor control	This suggests a penultimate period in which large-scale population-based studies are evaluating both the role of the biomarker in tumor detection and its ability to screen. These studies are designed to determine whether screening with a biomarker leads to a reduction in morbidity and mortality.

MicroRNAs (miRNAs) are short non-coding RNA molecules, approximately 18–22 nucleotides in length, that function as translation inhibitors by binding to their target mRNAs (mRNAs) in 3′-untranslated regions (3′-UTR) at the post-transcriptional level. To date, there are sufficient data on the physiological role of microRNAs, which play an important role in the control of the cell cycle, cell proliferation, differentiation and apoptosis. The levels of expression of aberrant miRNAs were also detected in the majority of tumor neoplasms. Based the level of expression of miRNAs and the main targets-oncogenes (or tumor suppressor genes), miRNAs can act as oncogenic miRNAs (onco-miRs) during tumor development and progression [1]. Onco-miRNAs, as a rule, are activated in normal cells under the influence of genetic damage, which, in turn, promote neoplastic transformation (tumorigenesis) by silencing tumor suppressor genes [2]. Given their important role in carcinogenesis, miRNAs themselves are also subject to control both at the post-transcriptional and epigenetic levels. Mutations in modifiers such as isocitrate dehydrogenase 1/2 (IDH1/2) and telomerase reverse transcriptase (TERT) lead to global changes in the epigenome, being common companions of glioma pathogenesis [2,3]. The roles that these mutations play in miRNA dysregulation and glioma development are unfortunately poorly understood. In this work, we will discuss the aberrant expression of miRNAs and their involvement in the development and progression of glial brain tumors. In addition, we will conduct a comprehensive review of miRNAs targeting a range of glioma traits, which include cell differentiation, apoptosis, angiogenesis, and invasion. We will also discuss the areas of application of miRNAs in the therapy of gliomas and the problems of their introduction into clinical activities.

## 2 The role of microRNA in tumor cell proliferation

The ability to continuously divide cells is a fundamental characteristic of all types of tumors, which are achieved by deregulating cellular signaling pathways. It is important to note that miRNAs can influence proliferation, as well as the ability to avoid tumor suppressors, increasing the ability of tumor cells to develop. This is illustrated by the ability of survival and proliferation under the control of epidermal growth factor receptor (EGFR) and Akt signaling pathway (RAC-alpha serine/threonine-protein kinase). In glial tumors, where increased EGFR expression is a characteristic feature of primary tumors, miRNAs that control EGFR expression reflect corresponding abnormalities and tumor progression. For example, miR-7, which acts as a tumor suppressor, directly targets EGFR and can independently suppress this signaling pathway. An increase in the expression of endogenous miR-7 in human glioblastoma cells allows activating the Akt signaling pathway and, thus, increasing the viability and invasiveness of tumor cells [4]. RAS proteins are also targets for miRNAs and play a key role in the deregulation of signaling pathways responsible for cell proliferation and differentiation in many tumors, including gliomas. Let-7 is a miRNA whose expression is decreased in glial cells and is inversely correlated with the presence of RAS proteins. This demonstrates that increasing the expression of let-7 reduces the effect of RAS, leading to a decrease in proliferation and migration of tumor cells in vitro and inhibition of tumor growth in vivo. However, let-7 had no effect on the proliferation of normal human astrocytes in vitro [5]. The Notch signaling pathway is an important regulator of cellular processes during the development of normal and tumor stem cells [6,7]. Activation of the Notch pathway enhances the proliferation and, interestingly, the radioresistance of tumor stem cells in gliomas [[6], [7], [8]]. It has been shown that downregulation of mir-34a expression in mature glioma cells and human stem cells inhibits the expression of c-Met (tyrosine-protein kinase Met), Notch-1, and Notch-2 by binding them to the 3′-UTR mRNA domains. This proves that miR-34a influences the proliferation, survival and migration of glial cells.

## 3 MiRNAs and apoptosis

In addition to tumor growth and abnormal cell proliferation, the ability to avoid apoptosis is an important characteristic of a tumor. Dysregulation of miRNAs expression is one of the mechanisms allowing tumor cells to bypass the pathways of programmed cell death. In addition, mediation between miRNAs and apoptosis is strongly associated with drug resistance, since many therapies are aimed at initiating apoptotic pathways. MiRNAs can have either pro- or anti-apoptotic functions and, therefore, differ in expression during tumor progression. Anti-apoptotic miRNAs target pro-apoptotic genes and are often found in glial tumors. MiR-21 is an anti-apoptotic miRNA that acts on transforming growth factor beta (TGF-β) and protein p53 [9]. Inhibition of miR-21 leads to activation of caspases, suppression of cell growth, a decrease in invasion, an increase in apoptosis, and an increase in chemosensitivity. These effects are partially mediated by a decrease in target repression, including heterogeneous nuclear ribonucleoprotein K (HNRPK), TAP63 and PDCD4 (Programmed cell death 4) [9,10]. In addition, miR-21 can modulate the external apoptotic pathway through suppression of FasL (Fas ligand), which is especially noticeable in tumor stem cells [11]. Thus, miR-21 has a broad influence on the pathways of apoptosis, making it an important component in the pathogenesis of gliomas and a promising target for therapy. Overexpression of miR-221 and miR-222 is manifested in glioblastoma cells, which have numerous targets involved in gliomagenesis, including cell apoptosis. MiR-221/222 can control apoptosis by targeting the pro-apoptotic protein p53 (PUMA, p53 upregulated modulator of apoptosis). Under normal conditions, PUMA is responsible for controlling apoptosis by binding to Bcl-2 (B-cell lymphoma 2) and Bcl-x (B-cell lymphoma 2). Thus, an increase in miR-221/222 expression and a subsequent decrease in PUMA expression promotes cell survival, and vice versa, a decrease in the expression levels of these miRNAs makes it possible to induce glial cell death and reduce tumor growth [12].

## 4 MiRNAs and angiogenesis

Angiogenesis is the formation of new blood vessels by remodeling pre-existing ones. One of the characteristic features of malignant gliomas is extensive neovascularization, where increased vascularization increases the proliferative and invasive capacity of tumor cells due to the greater availability of oxygen and nutrients. As the metabolic requirements of the growing tumor mass exceed the oxygen supply of the existing vascular network, creating conditions for hypoxia in the tumor tissues, the secretion of pro-angiogenic factors such as vascular endothelial growth factor (VEGF) begins. Elevated levels of VEGF and other pro-angiogenic factors lead to proliferation of endothelial cells and the formation of new blood vessels. This newly formed abnormal vasculature does not effectively supply tissues with a sufficient amount of oxygen, further contributing to hypoxia and thus resistance to therapy [13,14]. MiR-296 is one of the best studied miRNAs known to promote angiogenesis [[15], [16], [17]]. In one of the studies, the role of miR-296 in the angiogenesis of glial tumors was shown. It has been proven that overexpression of VEGF is capable of increasing the expression of endogenous miR-296 in human glioma cells in vitro. This indicates a reversal relationship by which VEGF induces the expression of miR-296, which in turn targets hepatocyte growth factor-regulated tyrosine kinase substrate (HGS), leading to increased levels of receptor-2 expression vascular endothelial growth factor receptor 2 (VEGFR2) and platelet growth factor -β receptor (PDGFR-β), thus increasing the expression of this miRNA in response to VEGF. In addition, VEGF is also capable of inducing an increase in miR-296 expression through a complex cross-talk mechanism with a growth factor receptor that increases miR-296 expression levels in a combinatorial manner [18]. The miR-17 and miR-93 family are activated in glioblastoma, increasing tumor cell survival, tumor growth, and neurosphere formation, in particular through angiogenesis. Overexpression of miR-93 induces the formation of new blood vessels, potentially by suppressing integrin-β8, a protein involved in cellular and cell-matrix interactions. Fang et al. found that vasculogenesis could be enhanced by overexpression of miR-93 in the human U87 glioblastoma cell line by co-cultured cells with endothelial cells [19]. This resulted in an increase in endothelial cell proliferation and tube formation in vitro and a high increase in blood vessel formation in glioblastoma xenograft tumors in mice [19]. These studies illustrate the important role that microRNAs play in the cross-talk mechanism between cells, acting as the most important regulators in the modulation of tumor cells and their microenvironment.

## 5 Tumor cell invasion and metastasis

High invasiveness of gliomas is the main factor of poor prognosis and treatment resistance in patients with this type of tumor. Unlike many other types of tumors, gliomas rarely metastasize through the vasculature or lymphatic vessels to other organs, but rather penetrate into the brain parenchyma itself. The reason for this is unclear, but may be associated with restrictions due to the blood-brain barrier (BBB) or neuron-specific microenvironment in the brain [18]. The main obstacle to the treatment of gliomas is the progressive penetration of tumor cells into deeper areas and their ability, thereby, to successfully avoid surgical treatment and radiation therapy. It is almost impossible to completely remove these diffusely penetrating cells by surgical resection [14,20]. The mechanisms of invasion in gliomas are poorly understood, and a better understanding of these mechanisms is necessary for the development of more effective treatments. Glial cells invading normal brain tissue tend to develop in a mesenchymal phenotype and migrate along blood vessels and white matter patches. These migrating cells mimic the migration of early progenitor cells during the development of the nervous system, i.e. epithelial-mesenchymal transition (EMT). The process of tumor cell invasion includes detachment of the interventional cell from the primary tumor mass, adhesion to the extracellular matrix (ECM) of normal tissue, and, finally, degradation and loss of connection with the ECM [18]. The changes in ECM provide the basic conditions for facilitating cell migration. One of the most common signaling pathways associated with cell migration and invasion is hepatocyte growth factor (HGF) and its receptor c-Met, which is one of the targets of miR-7 [21]. MiR-21 was the first miRNA to be found in glioblastoma cells in 2005 and is perhaps the most studied miRNA in these tumors to date. The activity of miR-21 is increased in cells and tissues of human gliomas in comparison with normal brain tissue [22]. In addition, miR-21 levels in gliomas correlate with tumor grade, and low miR-21 levels in human tumors are associated with better patient survival according to The Cancer Genome Atlas (TCGA) [23]. Among other cellular functions, miR-21 promotes invasion by directly acting on matrix metalloproteinases (MMPs) and proteolytic enzymes that degrade ECM. Decreased expression of MMP inhibitors, including reverse-inducing cysteine protein (RECK), myristolyated alanine C-kinase substrate (MARCKS), and tissue inhibitor of metalloproteinases-3(TIMP3), leads to activation of MMP and invasion. Studies have shown that inhibition of miR-21 leads to an increase in the expression of RECK and TIMP3, and, therefore, a decrease in MMP expression and glial cell invasion in a xenograft model of the glial cell line U87 [24]. MMPs are also targeted by other miRNAs, including miR-146b. It was shown that miR-146b inhibits the migration and invasion of glial tumor cells [25]. Using microarrays, miR-146b was identified as an aberrant miRNA in human glioblastoma cells. An increase in miR-146b expression did not affect the growth of human glioblastoma cells, but significantly reduced migration and invasion of one of the glioblastoma cell lines. MMP 16 has been identified as one of the downstream targets of miR-146b. Thus, the authors concluded that a decrease in MMP16 expression mediates the effects of miR-146b on invasion, but this has not been experimentally proven [25]. Numerous studies have shown that the level of miR-34a is reduced in glioblastoma cells compared to normal tissues [26,27]. Among other cellular functions, such as cell proliferation and survival, miR-34a reduces invasion in glioblastoma cells, in part by acting on HGF/c-Met and Notch1/2 signals [26]. The expression level of miR-10b is significantly increased in glioblastoma cells compared to normal brain tissue. The direct target of miR-10b, which is probably involved in the invasion of gliomas, includes Homeobox 10 (HOXD10), which negatively regulates the urokinase type plasminogen activator receptor (uPAR) and RhoC (RAS homolog family member C) [28]. An increase in miR-10b expression negatively affects the expression level of HOXD10 and positively affects the expression levels of RhoC and uPAR. These results indicate that this miRNA can control cellular invasion in the RhoC and uPAR-dependent mechanism [28]. Moreover, overexpression of miR-10b increases cell invasion, and its inhibition decreases cell invasion in vitro [29]. MiR-7 also has many targets involved in metastasis, including focal adhesion kinase (FAK), phosphoinositide 3-kinase (PI3K), EGFR, and RAF1 [4,30]. As indicated in its targets, miR-7 functions as a tumor suppressor and therefore is reduced in glioblastoma cells. Its overexpression can inhibit metastasis and invasion of glioblastoma cells by directly suppressing FAK, a mediator of extracellular matrix signaling, as well as by decreasing the expression of MMP2 and MMP9 [30]. In addition, by suppressing the expression of EGFR and inhibiting the Akt pathway, miR-7 can reduce the viability and invasiveness of glioblastoma cells [4].

## 6 MiRNAs as modulators of the effectiveness of chemotherapy and radiation therapy

Various drugs have been shown to alter miRNA expression in preclinical studies, suggesting that miRNAs may be suitable targets for anticancer agents. Expression of miRNA has been shown to alter the chemical sensitivity in glioblastoma. Overexpression of miR-21 significantly inhibited the effect of temozolomide (TMZ) on apoptosis, which was mediated by suppressing the expression of pro-apoptotic proteins Bax (BCL2-associated X protein) and caspase-3, as well as increasing the expression of the anti-apoptotic protein Bcl-2 [31]. MiR-21 targets LRRFIP1 (LRR Binding FLII Interacting Protein 1) and promotes teniposide resistance (VM-26) in glioblastoma [32]. MiR-21 enhances the cytotoxic effect of TMZ, doxorubicin, paclitaxel, and sunitinib in glioblastoma [33]. MiR-370-3p expression was suppressed in TMZ-resistant glioma cell lines. Expression of miR-370-3p increased the sensitivity of glioblastoma cells to TMZ by suppressing the self-reparative ability of DNA (deoxyribonucleic acid) of tumor cells. O-6-methylguanine-DNA methyltransferase (MGMT) was identified as an immediate putative target of miR-370-3p and increased expression of miR-370-3p restored glioblastoma sensitivity to TMZ, affecting MGMT expression [34]. Overexpression of miR-423-5p enhanced the ability of glioblastoma to form neurospheres and resulted in tumor cells becoming resistant to TMZ [35]. Accumulated data have shown that expression of miR-203 has been shown to enhance radio and chemosensitivity by suppressing EMT in glioblastoma [36]. Re-expression of miR-203 promoted an increase in the sensitivity of glioblastoma tumor cells to anticancer drugs and a decrease in cell invasion and migration. This study also demonstrated that miR-203 inhibited EMT and glioblastoma cell chemoresistance by targeting Snail Family Transcriptional Repressor 2 (SNAI2) [36]. Overexpression of miR-203 increased the sensitivity to radiation therapy of all three human glioblastoma cell lines [37]. This study also demonstrated that miR-203 potentially controls DNA damage repair via the PI3K/AKT (Phosphoinositide 3-kinases/AKT) and JAK/STAT3 (Janus Kinase 2/Signal transducer and activator of transcription 3) pathways and may collectively contribute to modulation of radiosensitivity in glioblastoma cells by inhibiting the repair of DNA damage and EMT [38]. Overexpression of miR-146b-5p increased the sensitivity to radiation therapy, thereby decreasing cell viability and the ability to form neurospheres [39].

## 7 The use of miRNAs in tumor therapy

MiRNAs are attractive candidates for the treatment of various diseases, including cancer, because of their small size, permanent class sequence, and relative stability. There are two general approaches to influencing miRNAs, which include miRNA agonists or mimics (miRNA mimics, agomirs) and miRNA antagonists or antagomirs (anti-miRs). Several approaches are proposed as therapeutic targets, where we will discuss the problems associated with their clinical use, including additional, “off-target” or “off-target” effect, tissue specificity, complications with cellular uptake and instability in vivo [40,41].

### 7.1 Strategic approaches

The use of anti-miRs is one of the approaches used to inhibit the function of the target microRNA. They consist of single-stranded RNA oligonucleotides that bind to a target and prevent the target microRNA from binding to its target mRNA. AMOs are a class of anti-miRs composed of chemically modified single-stranded oligonucleotides that irreversibly and specifically bind to complementary microRNA. The use of so-called miRNA-masking oligonucleotides (ONDs) is also one of the strategies in inhibiting miRNA function. The miRNAs masking strategy is used was called to inhibit the function of the target miRNA and includes masking the target site on the mRNA using a modified single-stranded RNA complementary to the target sequence [42]. Unlike antagomyrs, miRNAs are not degraded using this approach, so the corresponding function of a particular miRNA on other genes remains intact. Locked nucleic acid (LNA) blocking is a type of antagomir that involves the substitution of specific nucleotides with bicyclic RNA analogs in a fixed structure, which leads to higher similarity and better hybridization efficiency [43]. The disadvantages include their limited access to all tissues, the need for repeated administration in high doses to inhibit miRNA for a long period, and their tendency to accumulate in the liver [44]. Another representative of miRNA antagonists are the so-called miRNA sponges. Their role is also to prevent the binding of the target miRNAs with the target mRNA. Instead of separately targeting one miRNA, they can target all family members at once, since they recognize the same binding sequence [45]. The disadvantage is that “sponges” use competitive miRNAs that do not possess chemical modifications and, therefore, can be affected by low binding affinity and require a higher concentration to block the target [46]. In addition, there is a need for strong promoters and a need for multiple vector integration. MiRNA mimics are synthetic RNA duplexes in which one strand is identical to the mature miRNA sequence (guide strand) and is designed to “mimic” the function of the target miRNA. Another chain (passenger chain, guide strand) only partially complements the guide chain [40,41]. The double row structure is necessary for efficient recognition and loading of the guide strand in the RISC (RNA-induced silencing complex). For example, Chen et al. In their work found that the expression level of miR-203 is significantly reduced in glioblastoma compared to low-grade gliomas and normal brain tissue. Transfection with miR-203 mimics human glioblastoma U251 cells, which markedly suppresses the expression of phospholipase D2, which is a target of miR-213 and is believed to be oncogenic. This suppressed the proliferation and invasion of the human glioblastoma cell line U251, demonstrating the utility of miR-213 mimic in reducing the expression of target endogenous miR-213. Care must be taken when developing such therapies to rule out the potential danger of the passenger strand acting as a new miRNA and the possibility of causing unwanted side effects [47].

## 8 Delivery system

There are many studies showing good results in working with miRNAs in vitro, but studies with successful delivery of miRNAs in vivo are limited. Chemical modifications are often required to increase the stability of miRNA during its delivery, since, upon systemic administration, unmodified miRNAs can be degraded in the blood by nucleases or subsequently purified through renal secretion or the reticuloendothelial system [47]. In addition to chemically modified antagomir, the use of lenti- and adeno-associated viruses to deliver exogenous miRNAs has been reported in several studies [48,49]. Although modified adenoviruses or adeno-associated viral vectors can be effective for gene delivery, problems associated with the immune response to the virus are always of concern and are discussed in other experimental works. Thus, non-viral vectors that retain biocompatibility, targeting efficiency, and increased transfection efficiency are more suitable alternatives for achieving successful miRNA delivery without associated side effects. Mimics and antagomir can also be conjugated or complexed with nanoparticles, which makes them more resistant to nuclease degradation. Inorganic nanoparticles such as gold, silicon oxide and iron oxide are commonly used to deliver DNA. Gold is an inert element and does not react with most chemicals, making it useful for use in living organisms as a potential carrier for oligonucleotides. Recent studies have shown promising results that gold nanoparticles are able to penetrate the BBB in vivo [50]. It was shown that nanoparticles consisting of polyethylene glycol (PEG) -liposomal complexes provide similarity of miRNAs with low immunogenicity and long-term circulation. Using this approach, many studies have applied PEG - liposome complexes to liver target tumors [49]. For tumors of the central nervous system, the BBB represents a unique barrier for the delivery of microRNAs to the target tissue. Recent advances in research with drug delivery methods, including peptides and immunoliposomes, are now being revisited as new therapeutic approaches to bypass the BBB [51]. Even after miRNAs are successfully delivered to the tissues of interest, there is still the problem of accumulation of the target strain and possible side effects associated with exceeding doses of miRNA-associated therapy. In addition, various mechanical and biological barriers affect the delivery of microRNAs to specific target cells, including high interstitial pressure in tumor cells and the complexity of ECM [20,21].

## 9 Clinical trials

The main advantage of miRNAs is their ability to target multiple genes at once, and therefore they can effectively influence tumor heterogeneity. However, simultaneous targeting of multiple genes can also lead to unexpected side effects and unwanted toxicity. The main requirement for miRNA-associated therapies is the careful selection of the miRNA candidate. Ideally, microRNA should target the desired oncogene (s) with a minimum number of target mRNAs. In accordance with these measures, several miRNAs have successfully passed the preclinical stage and are discussed below. There are currently several ongoing clinical trials using miRNAs as tumor therapy. Unfortunately, there are no clinical trials of microRNA therapy for gliomas. To date, the first phase is the study of the drug Cobomarsen (MRG-106), which is used in the treatment of cutaneous T-cell lymphoma, which is a synthetic miRNA antagonist (LNA) that inhibits miR-155 [52]. Another is agonist therapy, where miR-16 is presented for patients with non-small cell lung cancer [52]. The means for delivery of miR-16 are “mini bacteria” or EnGeneIC delivery vehicles (EDV), the name is the same as for the manufacturer itself EnGeneIC [52]. One of the first proven methods of using miRNAs to treat cancer is MRX34, a synthetic miR-34a mimic with liposomes. It is known that miR-34a functions as a tumor suppressor. MRX34 directly inhibits at least 24 different oncogenes, including c-Met, Notch, CDK4, and BCL2. Preclinical results in mice have been promising and have shown successful, safe systemic delivery of miR-34a mimic without side effects from the immune system [53]. In 2013, a multicenter study was launched that included the treatment of patients with primary liver cancer, lymphoma, small cell lung cancer, and melanoma with miR-34a mimic administered by intravenous injection. Substantial evidence of antitumor activity and acceptable levels of safety was highlighted in a subgroup of patients with refractory progressive solid tumors [54]. However, the trial was suspended due to serious side effects related to the immune system, a massive release of cytokines. Currently, the trigger for these immune responses is unclear, and preclinical trials may need to be repeated [52]. That about neurotoxicity induced by microRNA-associated immunomodulation is an important area of research. MiRNA isolated from cancer cells can directly bind to Toll-like receptors (TLR) on the surface of neighboring immune cells, which can lead to the activation of unreasonable signaling pathways in recipient cells [55]. This can lead to neurodegeneration, which is evident during let-7b-mediated activation of Toll-like receptor 7 (TLR7) in neurons. Another effect on the immune system during MicroRNA therapy is the aberrant activation of specific innate immune effector cells, including natural killer cells (NK cells) along the TLR1-NF-kB pathway (nuclear factor kappa - B). This can affect multiple functions of NK cells, including cytokine production, proliferation, and cytotoxicity, which can alter the immune response and induce malignant transformation [55,56]. In addition, it can lead to the secretion of inflammatory cytokines and type I interferons (IFNs) by TLRs, depending on the structure, sequence and delivery systems of specific miRNAs, thus affecting the innate and adaptive immune response. This can activate a cascade of events leading to the priming of surrounding immune cells, as a result of which they become more sensitive to RNA stimulation [47,55,57]. These toxicity concerns need to be addressed in order to better understand and prevent immune-related side effects such as those experienced with MRX34. Another agent that has been used in the clinical field is miravirsen, where it is being evaluated in clinical trials for the treatment of hepatitis C virus (HCV) infection. Miravirsen is a β-D-hydroxy-LNA-modified oligonucleotide that targets miR-122. Specific miR-122 is endogenously expressed in hepatocytes and plays an important role in their development, differentiation, and metabolism. This miRNA is also involved in HCV RNA replication in a complex with the Argonaute 2 protein. This miR-122/viral RNA/Argonaute 2 protein complex also helps prevent HCV nucleolytic degradation. In the presence of miravirsen, miR-122 cannot bind to the complex, and the virus cannot replicate [58].

## 10 Conclusions

MiRNAs are believed to regulate the expression of one third of the human genome. Expression profiling of aberrant miRNAs provides a better understanding of glial tumor progression, providing valuable information about tumor pathogenesis and the potential use of miRNAs as biomarkers and therapeutic targets. MiRNAs positively or negatively regulate tumor cell proliferation, apoptosis, migration, invasion, angiogenesis, acting on numerous target genes. MiRNAs are also involved in the regulation of glioma malignancy and differentiation, indicating that deregulation of some miRNAs correlates with clinical prognosis. Despite the availability of reliable evidence that miRNAs are involved in the oncogenesis of glial tumors, the specific mechanisms of their participation are little known. Modern molecular biological research aimed at determining the targets of individual miRNAs and their clusters will undoubtedly allow in the future to achieve fine regulation of signaling pathways, violations of which are associated with neoplastic processes.

## Ethical approval

This article does not contain any studies with human participants performed by any of the authors.

## Author statement

Conceptualization: OB and FZ; Data curation: OB, AS and IG; Formal analysis: OB, AS, IG, TI, and FZ; Investigation: IG; Project administration: OB; Resources: OB and AS; Software: OB, FZ and IG; Supervision: OB; Validation: OB, TI and IG; Roles/Writing – original draft: OB and IG; Writing – review & editing: OB and FZ.

## Declaration of competing interest

The authors declare they have no conflict of interest.
